# SNP rs11185644 in RXRA gene and SNP rs2235544 in DIO1 gene predict dosage requirements in a cross-sectional sample of hypothyroid patients

**DOI:** 10.1186/s12902-023-01425-z

**Published:** 2023-08-10

**Authors:** Rowan AlEjielat, Anas Khaleel, Yazan S. Batarseh, Luay Abu-Qatouseh, Suzan Al-Wawi, Toleen AlSunna

**Affiliations:** 1 Faculty of Pharmacy and Medical Sciences, University of Petra, Amman, Jordan; 2 Independent researcher, Amman, Jordan

**Keywords:** Levothyroxine, SNP, Hypothyroid, Dose, Pharmacogenomics, Personalized medicine

## Abstract

**Background and purpose:**

Primary hypothyroidism due to abnormality in the thyroid gland is the most common endocrine disease The recommended starting dose of levothyroxine replacement therapy is 1.6 µg/kg. This dose however is not optimal for every patient and dose adjustments are frequently done. Genetic polymorphisms in the absorption and metabolism pathway of levothyroxine are likely to influence its dose requirements. This study aimed to study the influence of genetic polymorphisms on levothyroxine replacement requirements.

**Methods:**

This was a cross-sectional study. Participants were recruited through a private nutrition clinic and through announcements distributed in the University of Petra in Amman, Jordan between September 2020 and February 2021. Hypothyroid patients had already been on stable doses of levothyroxine for the previous 3 months. A questionnaire was distributed to collect demographic and clinical information and a blood sample was taken for DNA extraction and clinical biochemistry analysis. rs11249460, rs2235544, rs225014, rs225015, rs3806596, rs11185644, rs4588, rs602662 were analyzed using Applied Biosystems TaqMan™ SNP Genotyping Assays on Rotor-Gene® Q and rs3064744 by direct sequencing. SPSS and Excel were used to perform analysis.

**Results:**

76 patients were studied. The equation we calculated to find predicted daily dose of levothyroxine (mcg/kg) is 3.22+ (0.348 for CT genotype of rs11185644, 0 for other genotypes) + 0.027*disease duration (years) − 0.014*age (years) − 0.434*T3 (pmol/L) levels+ (0.296 for CC genotype of rs2235544, 0 for other genotypes).

**Conclusion:**

SNP rs11185644 in RXRA gene and SNP rs2235544 in DIO1 affect dose requirement in hypothyroid patients and if confirmed in larger trials they can be used to individualize thyroxine starting doses.

## Introduction

The most common endocrine disease is primary hypothyroidism, or thyroid hormone deficiency caused by a thyroid gland abnormality [1, 2]. The annual incidence of hypothyroidism is 4.1 per 1000 in women and 0.6 per 1000 in men [3]. Thyroid hormones exert significant influence over development and growth processes. In adults, their essential role lies in finely tuning the function and metabolism of virtually all organ systems [4–6]. Levothyroxine (LT4, L-thyroxine) is the preferred treatment for hypothyroidism because of its effectiveness in alleviating hypothyroid symptoms, extensive track record of benefits over time, favorable side effect profile, ease of administration, efficient intestinal absorption, extended serum half-life, and affordability [5, 6]. In primary hypothyroidism, the objectives of LT4 replacement therapy are to attain a state of euthyroidism and restore normal levels of circulating thyroid hormones and TSH [5].

The daily dosage of levothyroxine is dependent on age, sex, and body size [6]. Dosage considerations should also be given to additional factors such as pregnancy status, the underlying cause of hypothyroidism, the extent of thyrotropin elevation, and the overall clinical context, which sometimes includes the presence of cardiac disease [5]. The recommended starting dose of LT4 is 1.6 µg/kg but this dose is not optimal for every hypothyroid patient and dose changes are frequently done to reach optimum treatment [5–8].

TSH serves as the most dependable indicator of the adequacy of replacement treatment, and it is recommended to maintain a value within the reference range (0.4–4.0 mIU/L) [5]. But to monitor for treatment sufficiency, the patient must wait around 5–6 weeks after initiation of therapy or change of dose to check for optimal treatment. This is due to the relatively extended half-life of LT4, which lasts around 7 days. As a result, it takes about 6 weeks (between five to seven half-lives) after starting the therapy to reach steady-state levels of T4 and TSH [5]. This essentially means that most patients will need to wait a few months to reach optimum treatment doses, which may be inconvenient for them and have an impact on their quality of life.

Other than the above-mentioned traditional influencers of levothyroxine dosing, many genetic factors are likely to also interfere with LT4 dose requirements. These can include polymorphisms in the absorption and metabolism pathway of levothyroxine. Thyroid hormones undergo metabolism through various pathways, including glucuronidation, sulfation, and deiodination. Among these, deiodination is the most significant. Deiodination involves three specific enzymes known as type 1 (D1), type 2 (D2), and type 3 (D3) iodothyronine deiodinases. For example, polymorphisms in the transporters that are responsible for absorption can affect the serum level of the hormone after drug administration, other likely genetic determinants are polymorphisms in the deiodinases which convert LT4 to the active form T3. As well as polymorphisms in the metabolic enzymes Glucuridine-transferases and sulfases [4, 9–16].

There were a few studies from Jordan which examined the effect of polymorphisms in genes correlating to LT4 but they were only a single gene or two genes, none of them have attempted to study polymorphisms in multiple genes across the metabolism pathway and none have tried to create a new model for dosing [15, 17]. In this current study, we characterized the effect of candidate single nucleotide polymorphisms (SNPs) that were significantly correlated with LT4 serum levels from previous literature in combination with the effects of new candidate SNPs as well as traditional dosing predictors to try to create a more accurate dosing model for LT4 which will shorten time to reach adequacy of treatment. A model will likely help cases that are sensitive such as patients with heart disease, and pregnant women although they were not included in this study.

The main objective of this study was to screen and describe the frequency of pre-defined candidate polymorphisms in the genetic pathway of levothyroxine in hypothyroid patients who were being treated in Jordan. The secondary objective was to develop a new model/formula for calculating a levothyroxine starting dose in hypothyroid patients, taking into consideration traditional factors as well as genetic polymorphisms. Our research was mainly exploratory and attempted to describe the feasibility of these types of translational studies.

## Methods

### Participants

This was a cross-sectional study approved by the ethical committee of The University of Petra (1 (H/9/2020) and was carried out in accordance with the Helsinki Declaration of 1975, with all amendments and revisions. Participants were recruited through a private nutrition clinic in Amman, Jordan and through announcements distributed in the University of Petra between September 2020 and February 2021. Hypothyroid patients were past puberty and had already been on stable doses of L-T4 for the previous 3 months. Those with active neoplasm or a history of neoplasm, severe liver dysfunction, severe-renal failure, pituitary gland or hypothalamic diseases, including secondary hypothyroidism, major surgery within 2 weeks of enrollment, a severe psychiatric condition unrelated to hypothyroidism symptoms, GIT malabsorption diseases, pregnancy, alcohol abuse, and concurrent medication that may interfere with thyroid hormone absorption or activation were excluded from this study. Patient consent was obtained in writing, and a questionnaire was filled out for each participant. The questionnaire collected information on Age, Gender, Height, weight, waist circumference, type and dose of thyroid hormone replacements, and time of administration and adherence, current and past medical history, family history of thyroid and other diseases and smoking status.

### Blood sample collection

6 ml of blood was taken and divided into two tubes one for clinical biochemistry and one for DNA extraction and genotyping and then frozen in -80 freezer till analysis.

### Clinical biochemistry

TSH, fT3, fT4 ,TPO, B12 and Vitamin D levels were measured in a private lab using the Cobas e411 analyzer from Roche. Calcium levels were measured using the Cobas C111 analyzer.

### DNA extraction and quantitation

3 ml of peripheral blood from the patients was collected in EDTA coated Vacutainer for DNA isolation. QIAamp DNA Blood Mini Kits from Qiagen® were used for DNA extraction according to manufacturer’s protocol. DNA quantity and purity was assessed using the NanoDrop 2000/2000c Spectrophotometer from Thermo Scientific®.

### Genotyping

The following SNPs were extracted from literature: rs11249460, rs2235544, rs225014, rs225015, rs3806596, rs11185644, rs4588, rs602662, rs3064744.

They were selected to represent different parts of the metabolic pathway of the thyroid hormone. These were also selected based on having a reasonable expected minor allele frequency.

All SNPs except for rs3064744 were genotyped using Applied Biosystems TaqMan™ SNP Genotyping Assays and Qiagen’s Type-it® Fast SNP Probe PCR Kit supplied from ThermoFisher Scientific on the Rotor-Gene® Q from Qiagen®.

Genotyping conditions for Rotor-Gene® Q were as follows: Hot start for 5 min at 95 ° C followed by two-step cycling for 45 cycles: denaturation at 95 ° C for 15 s then annealing/extension at 60° C for 30 s.

The UGT1A1 rs3064744 was sequenced directly from PCR product after purification [18]. PCR was performed on GeneAmp® PCR system 9700 from Applied Biosystems. Biorad Gel Doc EZ imager was used for visualization of PCR products on 2% agarose gel. A 404-bp PCR fragment was amplified using the primers forward UGT1A1F 5´ gaggttctggaagtactttgc 3´ and reverse UGT1A1R 5´ ccaagcatgctcagccag 3´. PCR was performed using OneTaq® Quick-Load® 2X master mix with standard buffer NewEngland-Biolabs®. The 50 µl final mix for PCR constituted of 25 µl of master mix, 1 µl of each primer, 1 µl template DNA and DNAs free water up to 50 µl. PCR conditions: Initial Denaturation at 95 ° C for 5 min followed by 30 cycles of denaturation at 95° C for 1 min, then annealing at 58° C for 30 s and extension at 72° C for 30 s with a final hold at 4 ° C. PCR products were purified using the DNA clean and concentrator TM -5 from Zymo Research® and stored at -20 ° C till pick up. Purified products were sent to CD Genomics/USA for direct sequencing. Genotype was assigned by visual check of the raw data of the sequence reads on SnapGene viewer 6.1.1 by two separate researchers.

Linkage Disequilibrium results were calculated from the online Calculator from Ensembl based on GRCh37 Release 108 (Oct 2022) TSI population, detailed results can be found on https://grch37.ensembl.org/Homo_sapiens/Tools/LD/Results?tl=4OdB02whASvBsdY2-8834410.

### Statistical analysis

IBM SPSS statistics version 25 and Excel for Microsoft 365 were used to perform analysis. Means and percentages or means and ranges were used to describe demographics. Chi-square test (CHITEST, on excel) was used to test for Hardy-Weinberg equilibrium. Pearson Correlations were calculated using SPSS. ANOVA was used to test for the significant effect of different genotypes on the various variables. Normality of data was checked visually. Levene’s test was used to check for equal variance before running ANOVA. Welch’s test was used when equality of variance was not met. Post hoc Tukey HSD was used for pairwise comparisons. The false discovery rate (FDR) method, provided through the Q value package was used with RStudio 2022.12.0 Build 353, Posit Software, PBC, to adjust for multiple testing (R package version 2.30.0) [19].

Linear regression function from the SPSS software was used to predict T4, T3, TSH, weight adjusted daily dose levels in four different models. Antithyroid peroxidase antibodies were studied as categorical variable the cut-off value of 34 was used to indicate presence or absence of Autoimmune Hashimoto Thyroiditis, this value was the cut off obtained from the lab analysis.

## Results

93 Patients were recruited. Clinical Chemistry and genotype analysis was completed for all 93 patients. Out of these 93 patients 17 were found to have out of range TSH values, i.e., their disease was uncontrolled and were discovered randomly. These patients were excluded from further analysis and model building.

The stable TSH cohort consisted of 76 participants. Demographic data for the full cohort and stable cohort are shown in Table 1. Our sample consisted of mainly females (92.5%). All were of middle eastern descent; hence the sample was considered homogenous. The mean age was 46.45 years with a range from 21to 83 years. Participants were all from urban cities in Jordan.

**Table 1:** Demographics N = 93. FC: Full Cohort. SC: Stable Cohort where TSH is within normal range

Parameter	FC (N = 93)	SC (N = 76)
Female, N (%)	86 (92.5%)	72(94.7%)
Male, N (%)	7 (7.5%)	4 (5.3%)
Age, Mean(range)	44.7 (19–83)	46.45 (21–83)
Weight, Mean(range)	75.6 (46.3-129.1) *missing = 2	75.5 (46.3-129.1) *missing = 1
Height, Mean(range)	162.1 (149–187) *missing = 1	162.1 (149–187)
Waist circumference, Mean(range)	97.34 (74–159) *missing = 1	97.9 (74–159) * *Missing = 16
BMI, Mean(range)	28.8 (18.3–45.7) *missing = 2	28.8 (18.4–45.7) *Missing = 1
Nationality, N (%)	Jordanian 90 (96.8%) Iraqi 2 (2.2%) Syrian 1 (1.1%)	Jordanian 75 (98.7%) Iraqi 1 (1.3%)
Disease Duration (years)	8 (0.3–32) *Missing = 5	8.5 (0.3–32) *Missing = 5
Living area, N (%)	Amman 82(88.2%) Madaba 3(3.2%) AlKarak 2(2.2%) Ajloun 2(2.2%) AlMafraq 1(1.1%) AlFuhais 1(1.1%) AlZarqa 1(1.1%) AlSalt 1(1.1%)	Amman 67(88.2%) Madaba 3(3.9%) AlKarak1(1.3%) Ajloun 1(1.3%) AlMafraq 1(1.3%) AlFuhais 1(1.3%) AlZarqa 1(1.3%) AlSalt (1.3%)
Weekly dose T4 (µg), Mean(range)	612.903(75-2500)	599.3 (175–2500)
Daily dose T4 (µg), Mean(range)	87.56 (10.71-357.14)	85.6 (25–357)
Weekly dose (µg) per kg body weight, Mean(range)	8.1 (1.27–28.54) *missing = 2	8.14 (1.54–28.54) *Missing = 1
Daily dose (µg) per kg body weight, Mean(range)	1.15 (0.18–4.08) *missing = 2	1.16 (0.22–4.08) *Missing = 1
TSH mean (range)	2.38 (0.02–37.32)	1.73 (0.29–4.24) **Within Lab normal
Free T4 serum level pmol/l	19.4 (8.29–34.49)	19.06 (10.2-27.03)
Free T3 serum level pmol/l	4.51 (2.61–6.39)	1.74 (3.19–6.14)
Antithyroid peroxidase antibodies (AntiTPO)	132.5 (< 9->600)	118.8 (9-600)
AntiTPO (*34 cut off of positivity)	< 34: 46 (49.5%)	< 34: 30 (50%)
	> 34: 47(50.5%)	> 34: 30 (50%)
Serum Calcium mmol/L	2.34 (2.08–2.85)	2.4 (2.08–2.85)
Serum Vitamin D nmol/L	70.73 (14.53–190.00)	73.25 (17.5–190)
Serum B12 pmol/L	343.18 (71-2000)	353.9 (71-2000)
B12 supplementation	Yes: 11 (11.8%) No: 82 (88.2%)	Yes: 9(11.8%) No: 67(88.2%)
Vitamin D supplementation	Yes: 45 (48.4%) No: 48 (51.6%)	Yes: 36(47.4%) No: 40(52.6%)
Calcium supplementation	Yes: 7 (7.5%) No: 86 (92.5%)	Yes: 6(7.9%) No: 70(92.1%)
Other supplements	Yes: 29 (31.2%) No: 64 (68.8%)	Yes: 23(30.3%) No: 52(68.4%)
Family history of Thyroid disease	No: 31 (33.3%) Yes: 62 (66.7%)	No: 24(31.6%) Yes: 52(68.4%)
Adherence	Full adherence: 90 (96.8%) 1 miss per month: 1 (1.1%) 2 miss per month: 1 (1.1%) 3 miss per month: 1 (1.1%)	Full adherence:74 (97.4%) 1 miss per month: 1(1.3%) 3 misses per month: 1(1.3%)
Smoking status	Nonsmoker: 69(74.2%)	Nonsmoker: 57 (75.0%)
	Smoker: 20(21.5%)	Smoker: 16(21.1%)
	Quit all types: 4(4.3%)	Quit all types: 3(3.9%)

*Missing values: patients refused to give weight or waist circumference measurements after initial consent to participate. Disease duration missing values were due to errors in data collection. All these values were considered to be missing at random.

All SNPs were under Hardy Weinberg Equilibrium (HWE) for both the full cohort (data not shown) and for the stable cohort. Table 2 displays the allele and genotype frequencies for SNPs in addition to the p-Value for HWE Chi-square for the stable cohort.

**Table 2:** Genotype and allele frequencies and HWE for stable cohort (N = 76)

Polymorphism	Genotype	(N)%	Allele	(N)%	HWE (Χ^2^ P value)
SULT1B1 rs11249460	AA	9(11.8)	A	57(37.5)	0.7100
	AG	39(51.3)	G	95 (62.5)	
	GG	28(36.8)			
DIO1 Rs2235544	AA	14(18.4)	A	60(39.5)	0.5840
	AC	32(42.1)	C	92(60.5)	
	CC	30(39.5)			
DIO2 Rs225014	CC	10(13.2)	C	52(34.2)	0.8533
	CT	32(42.1)	T	100(65.8)	
	TT	34(44.7)			
DIO2 Rs225015	AA	8(10.5)	A	48(31.6)	0.9753
	AG	32(42.1)	G	104(68.4)	
	GG	36(47.4)			
UGT1A4 Rs3806596	CC	32(42.1)	C	98(64.5)	0.9794
	CT	34(44.7)	T	54(35.5)	
	TT	10(13.2)			
VD rs11185644	CC	7(9.2)	C	42(27.6)	0.7899
	CT	28(36.8)	T	110(72.4)	
	TT	41(53.9)			
VD Rs4588	GG	40(52.6)	G	113(74.3)	0.4840
	GT	33(43.4)	T	39 (25.7)	
	TT	3(3.9)			
B12 (FUT2) Rs602662	AA	27(35.5)	A	94(61.8)	0.6040
	AG	40(52.6)	G	58(38.2)	
	GG	9(11.8)			
UGT1A1 (rs3064744)	*1/*28	43(56.6)	*1(6 repeats)	76(50)	0.0000
	*1/*36	33(43.4)	*28(7 repeats)	43(28.3)	
			*36(5 repeats)	33(21.7)	

* Equals number of TA repeats in the promoter region: *1 (6 repeats), *28 (7 repeats), *36 (5 repeats) and *37 (8 repeats)

### Linkage disequilibrium results

Only two SNPs were in high linkage with each other: rs225015 with rs225014 (r^2^: 0.9, D’: 1), two other pairs were linked but not to a significant extent. rs11249460 with rs11185644 (r^2^: 0.05, D’: 0.32) and rs2235544 with rs3806596 (r^2^: 0.07, D’: 0.28).

### Association of SNP genotypes with different thyroid function parameters

Table 3 portrays the mean values of different thyroid function parameters across the genotypes of the selected SNPs. We saw a statistically significant difference in the levels of TSH, T3 and T4, dose, as well as BMI and age across the different genotypes of these variations. FDR cut off point was set to 0.2 because our study was mainly exploratory. After correction, only 3 results remained statistically significant (I.e., q value < 0.2). The full results are described below.

**Table 3:** ANOVA table across genotypes for stable cohort

Polymorphism	Genotype	N	Conc T4/dose (mean)	P value, q value	Weekly dose	P value, q value	Daily dose	P value, q value	Weekly dose/kg	P value, q value	Daily dose/kg	P value, q value	TSH	P value, q value	T4 pmol/l	P value, q value	T3 pmol/l	P value, q value	BMI	P value, q value
rs11249460	AA	9	2.04	0.766 0.891	712.5	0.363 0.253	101.79	0.363 0.253	8.62	0.556 0.412	1.23	0.556 0.412	2.44	0.045# 0.757	19.62	0.642 0.757	4.28	0.585 0.834	33.87	0.02# 0.795
	AG	39	1.65		614.1		87.73		8.50		1.21		1.73		18.74		4.34		28.09	
	GG	27	1.58		542.4		77.49		7.45		1.06		1.52		19.34		4.47		28.05	
rs2235544	AA	14	2.03	0.408 0.800	516.07	0.485 0.873	73.72	0.485 0.873	7.11	0.191 0.757	1.02	0.191 0.757	1.87	0.780 0.938	18.91	0.972 0.978	4.36	**0.002#** 0.108*	28.64	0.012# 0.243
	AC	30	1.51		642.50		91.79		9.20		1.31		1.65		19.16		4.15		26.47	
	CC	32	1.66		595.31		85.04		7.63		1.09		1.76		19.05		4.64		30.92	
rs225014	CC	10	1.26	0.074 0.500	652.50	0.536 0.891	93.21	0.536 0.891	9.02	0.406 0.800	1.29	0.406 0.800	1.58	0.678 0.938	19.25	0.938 0.970	4.25	0.713 0.938	30.10	0.725 0.938
	CT	32	1.99		551.17		78.74		7.40		1.06		1.85		19.16		4.39		28.35	
	TT	34	1.51		629.04		89.86		8.55		1.22		1.68		18.92		4.42		28.76	
rs225015	AA	8	1.30	0.415 0.800	618.75	0.799 0.938	88.39	0.799 0.938	8.91	0.594 0.891	1.27	0.594 0.891	1.85	0.946 0.970	19.26	0.978 0.978	4.30	0.863 0.970	29.51	0.925 0.970
	AG	32	1.87		569.92		81.42		7.58		1.08		1.73		19.00		4.37		28.80	
	GG	36	1.59		621.18		88.74		8.45		1.21		1.72		19.08		4.41		28.59	
rs3806596	CC	32	1.65	0.407 0.800	624.22	0.295 0.771	89.17	0.295 0.771	8.24	0.270 0.757	1.18	0.270 0.757	1.69	0.186 0.757	19.65	0.018# 0.253	4.24	0.086 0.536	29.96	0.271 0.757
	CT	34	1.83		542.65		77.52		7.51		1.07		1.63		19.27		4.54		28.20	
	TT	10	1.25		712.50		101.79		9.90		1.41		2.26		16.49		4.29		26.85	
rs3064744	*1/*28	43	1.81	0.261 0.757	602.03	0.935 0.970	86.00	0.935 0.970	7.76	0.360 0.795	1.11	0.360 0.795	1.69	0.652 0.938	20.00	**0.003#** 0.108*	4.31	0.218 0.757	30.42	**0.004#** 0.108*
	*1/*36	33	1.49		595.83		85.12		8.64		1.23		1.79		17.85		4.47		26.56	
rs11185644	CC	7	1.79	0.557 0.891	521.43	0.025# 0.253	74.49	0.025# 0.253	7.62	0.056# 0.412	1.09	0.056# 0.412	1.43	0.166 0.757	20.35	0.237 0.757	4.60	0.443 0.834	26.71	0.335 0.795
	CT	28	1.48		729.91		104.27		9.59		1.37		2.01		19.52		4.31		29.95	
	TT	41	1.79		523.48		74.78		7.21		1.03		1.60		18.53		4.40		28.31	
rs4588	GG	40	1.75	0.861 0.970	540.00	0.208 0.757	77.14	0.208 0.757	7.27	0.131 0.707	1.04	0.131 0.707	1.83	0.703 0.938	19.04	0.878 0.970	4.42	0.697 0.938	29.04	0.839 0.970
	GT	33	1.60		674.24		96.32		9.21		1.32		1.63		19.17		4.36		28.35	
	TT	3	1.57		566.67		80.95		7.58		1.08		1.65		18.19		4.16		29.91	
rs602662	AA	27	1.75	0.578 0.891	537.04	0.267 0.757	76.72	0.267 0.757	7.66	0.342 0.795	1.09	0.342 0.795	1.74	0.711 0.938	18.85	0.897 0.970	4.32	0.778 0.938	28.06	0.749 0.938
	AG	40	1.72		610.62		87.23		8.04		1.15		1.68		19.15		4.41		29.19	
	GG	9	1.28		736.11		105.16		9.96		1.42		1.98		19.35		4.45		29.00	

# significant p value at the level of < 0.05, * significant after correction for multiple testing, FDR of 0.2

Doses are in mcg or mcg/kg. Normality of data was checked visually. Levene’s test was used to check for equal variance before running ANOVA. Welch’s test was used when equality of variance was not met. Post hoc analysis results of Tukey HSD: SULT1B1 rs11249460 AA Vs. GG only (P = 0.035) for TSH and both AA Vs. GG and AA Vs. AG (P = 0.026, 0.02 respectively) for BMI. DIO1 rs2235544: AC Vs. CC only for both BMI (P = 0.008) and T3 (P = 0.001). UGT1A4 Rs3806596: TT Vs. CC (P = 0.014) and TT Vs. CT (P = 0.035) for T4. VD rs11185644: CT Vs. TT (P = 0.023) for both weekly dose and daily dose

The mean levels of TSH where higher in the AA genotype of rs11249460 in SULT1B1 (Mean: 2.44, 95% CI:1.589–3.297) compared to the GG genotype (Mean: 1.52, 95% CI: 1.195–1.841) post hoc p-value = 0.035, q-value = 0.757. In addition, the BMI was higher in the AA genotype of rs11249460 (33.87, 95% CI: 29.14–38.61) compared to the GG (28.05, 95% CI: 26.14–29.97) and GA (28.09, 95% CI: 26.11–30.06) genotypes, post hoc p-values = 0.026, 0.02 respectively, q-value = 0.795.

The mean levels of T3 AC genotype were lower in the DIO1 rs2235544 (Mean: 4.15, 95% CI: 4.00-4.30) compared to the CC genotype (Mean: 4.64, 95% CI: 4.42–4.86), post hoc p-value = 0.001, q-value = 0.108*. But the mean levels of BMI for the AC genotype were higher in the same SNP rs2235544 (Mean: 30.92, 95% CI: 28.80- 33.04) compared to the CC genotype (Mean: 26.47, 95% CI: 24.42–28.51), post hoc p-value = 0.008, q-value = 0.243. On the other hand, the mean T4 levels of the TT genotype of the rs3806596 in the metabolizing enzyme UGT1A3 (mean: 16.49, 95% CI: 14.24–18.74) was lower compared to the CC genotype (mean: 19.66, 95% CI: 18.55–20.76) post hoc p-value = 0.014 and CT (mean: 19.27, 95% CI: 18.23–20.30) post hoc p-value = 0.035 genotypes, q-value = 0.253.

The genotypes within rs3064744 were significantly different after correction. Mean T4 levels were higher in *1/*28 genotype (Mean: 20, 95% CI: 19.07–20.93) compared to the *1/*36 (Mean: 17.85, 95% CI: 16.79–18.90), p-value = 0.003, q-value = 0.108*. Mean BMI levels were higher in *1/*28 genotype (Mean: 30.42, 95% CI: 28.63–32.21) compared to the *1/*36 (Mean: 26.56, 95% CI: 24.62–28.49), p-value = 0.004, q-value = 0.108* .

Interestingly, both the mean weekly dose (mean: 729.91, 95% CI: 562.20-897.62) and daily dose (mean: 104.27, 95% CI: 80.31-128.23) for the CT genotype of SNP rs11185644 in RXRA gene were higher than the TT genotype; weekly dose (mean: 523.48, 95% CI: 457.41-589.55) post hoc p-value = 0.023, q-value = 0.253 and daily dose (mean: 74.78, 95% CI: 65.34–84.22) post hoc p-value = 0.023, q-value = 0.253. Post hoc analysis was not significant for the CC genotype although as a value it was close to the CT genotype. For the weight adjusted doses, the test was not significant (p-value = 0.056).

### Linear regression models to predict daily-dose/weight, T3, T4 and TSH levels

We performed linear regression analysis to find predictors of weight adjusted daily dose. We included several variables in the model to control for their effect including all the SNPs, sex, age, TPO status (coded as categorical positive or negative), adherence, smoking status, T3, TSH, T4, supplementation status of VD, supplementation status of B12 and other supplements, family history, vitamin D blood level, B12 blood level, Ca^+ 2^ blood level (Tables 4, 5, 6 and 7). We found the following equation to calculate weight adjusted dose for people 18-year-old and above: predicted daily dose (mcg/kg) = 3.22+ (0.348 for CT genotype of rs11185644, 0 for other genotypes) + 0.027*disease duration (years) − 0.014*age (years) − 0.434*T3 (pmol/L) levels+ (0.296 for CC genotype of rs2235544, 0 for other genotypes).

**Table 4:** Linear regression analysis to find independent predictors of dose/weight level in hypothyroid patients treated with Levothyroxine. Only statistically significant variables are presented

Variable	Coefficient	95% Confidence interval		P Value
		Lower bound	Upper bound	
(Constant)	3.222	1.961	4.483	0.000
rs11185644 CT Vs other genotypes	0.348	0.103	0.593	0.006
Disease Duration (years)	0.027	0.007	0.047	0.008
Age for DOB	-0.014	− 0.023	-0.005	0.003
Free Triiodothyronine (FT3), serum level pmol/L SI UNIT	-0.434	-0.678	-0.190	0.001
rs2235544 CC VS. Other genotypes	0.296	0.035	0.557	0.027

R square = 0.368, P value = 0.000

Criteria: Stepwise (Criteria: Probability-of-F-to-enter < = 0.050, Probability-of-F-to-remove > = 0.100).

Equation to calculate predicted dose (mcg/kg) levels = 3.22+ (0.348 for CT genotype of rs11185644) + 0.027*disease duration- 0.014*age- 0.434*T3 levels+ (0.296 for CC genotype of rs2235544)

All Variables studied: All 8 SNPs, sex, age, TPO status, adherence, smoking status, T3, TSH, T4 Supplementation status of VD, B12 and other supplements, family history, vitamin D level, b12 level, Ca level

**Table 5:** Linear regression analysis to find independent predictors of T3 level in hypothyroid patients treated with Levothyroxine. Only statistically significant variables are presented

Variable	Coefficient	95% Confidence interval		P Value
		Lower bound	Upper bound	
(Constant)	3.674	2.873	4.474	0.000
Age for DOB	-0.013	-0.019	-0.006	0.000
Weekly dose of thyroxine (mg)	-0.001	-0.001	0.000	0.002
DIO1Rs2235544 CC VS. Other genotypes	0.375	0.165	0.584	0.001
Other supplements	0.345	0.125	0.565	0.003
Sex	0.493	0.065	0.921	0.025
Supplementation of VD	0.210	0.009	0.411	0.041

R square = 0.525, P value = 0.000

Criteria: Stepwise (Criteria: Probability-of-F-to-enter < = 0.050, Probability-of-F-to-remove > = 0.100).

Predicted T3 levels = 3.674 − 0.013*Age- 0.001*weekly dose of thyroxine +(0.375 For CC genotype of rs2235544) + 0.345* supplement status (1 for yes, 0 for No) + 0.493 (sex) + 0.21* supplementation status of VD (1 for yes, 0 for No)

All Variables studies: All 8 SNPs, sex, age, BMI, TPO status, adherence, smoking status, T3, TSH, Supplementation status of VD, B12 and other supplements, family history, vitamin D level, b12 level, Ca level

**Table 6:** Linear regression analysis to find independent predictors of T4 level in hypothyroid patients treated with Levothyroxine. Only statistically significant variables are presented

Variable	Coefficient	95% Confidence interval		P Value
		Lower bound	Upper bound	
(Constant)	18.911	17.081	20.741	0.000
UGT1A3rs3806596 TT VS. other genotypes	-3.281	-5.292	-1.271	0.002
Weekly dose of thyroxine (mg)	0.003	0.001	0.005	0.004
Thyroid Stimulating Hormone (TSH), Serum mU/L SI UNIT	-0.771	-1.461	-0.082	0.029

R square = 0.311, P value = 0.000

Criteria: Stepwise (Criteria: Probability-of-F-to-enter < = 0.050, Probability-of-F-to-remove > = 0.100).

Equation to calculate Predicted T4 levels = 18.911- (3.281 For TT genotype of rs3806596) + 0.005* weekly dose of thyroxine − 0.771* TSH level

All Variables studied: All 8 SNPs, sex, age, BMI, TPO status, adherence, smoking status, T3, TSH, Supplementation status of VD, B12 and other supplements, family history, vitamin D level, b12 level, Ca level

**Table 7:** Linear regression analysis to find independent predictors of TSH level in hypothyroid patients treated with Levothyroxine. Only statistically significant variables are presented

Variable	Coefficient	95% Confidence interval		P Value
		Lower bound	Upper bound	
(Constant)	3.896	2.583	5.208	0.000
Free Thyroxine (FT4), serum level pmol/L	-0.124	-0.193	-0.055	0.001
VDrs11185644CT Vs other genotypes	0.547	0.104	0.990	0.016

R square = 0.203, P value = 0.001

Criteria: Stepwise (Criteria: Probability-of-F-to-enter < = 0.050, Probability-of-F-to-remove > = 0.100).

Equation to calculate Predicted TSH levels = 3.896 − 0.124* T4 level + (0.547 for CT genotype of rs11185644)

All Variables studies: All 8 SNPs, sex, age, BMI, TPO status, adherence, smoking status, T3, TSH, Supplementation status of VD, B12 and other supplements, family history, vitamin D level, b12 level, Ca level

We also performed three other linear regression analyses to find factors the predict/influence T3 T4, and TSH levels. For all of these, the following predictors were studied: All 8 SNPs, sex, age, BMI, TPO status, adherence, smoking status, Supplementation status of VD, B12 and other supplements, family history, vitamin D blood level, B12 blood level, Ca^+ 2^ blood level and lastly T3 or TSH or T4, depending on what we are predicting, the other two will be included. Tables 4, 5, 6 and 7 show 4 linear regression models to predict dose/weight, T3, T4, and TSH values. For T4 we found these predictors: UGT1A3 rs3806596, Weekly dose of thyroxine (mcg) and TSH with the following predicting equation: Predicted T4 levels = 18.911- (3.281 For TT genotype of rs3806596, 0 for other genotypes) + 0.005* weekly dose of thyroxine − 0.771* TSH level.

For T3 we found these predictors: Age, weekly dose of thyroxine (mcg), DIO1 rs2235544, Other supplements, sex, supplementation of VD with the following predicting equation: Predicted T3 levels = 3.674 − 0.013*Age (years)- 0.001* weekly dose of thyroxine (mcg)+(0.375 For CC genotype of rs2235544, 0 for other genotypes) + 0.345* supplement status (1 for yes, 0 for No) + 0.493 sex (1 female, 0 male) + 0.21* supplementation status of VD (1 for yes, 0 for No).

And lastly for TSH we found these predictors: Free T4 serum level pmol/L, rs11185644, with the following predicting equation: Predicted TSH levels = 3.896 − 0.124* T4 level + (0.547 for CT genotype of rs11185644).

## Discussion

In this cross-sectional study of hypothyroid patients, we have described the frequency of alleles and genotypes of 8 selected candidate SNPs that were identified to be part of the metabolic pathway of levothyroxine. To our knowledge this is the first study to report the genetic profiles for these selected SNPs in the Jordanian Population.

SULT1B1 (Sulfotransferase Family 1B Member 1) is a phase II drug-metabolizing enzyme. Sulfation by sulfotransferase enzymes (SULTs) is an important pathway for the metabolism of thyroid hormones and phytoestrogens [20]. rs11249460 in this gene was associated previously with SULT1B1 abundance levels. The TT genotype being associated with less abundant levels compared to the CT genotype and the latter less than the CC genotype [21]. In our initial analysis the TT or (AA) genotype was associated with higher TSH levels. Interestingly BMI levels were also affected by the rs11249460 in this gene in the same direction in the initial analysis. After correction for multiple testing both were not statistically significant. SULT1B1is not one of the extensively studied genes in relation to the thyroid pathway and its detailed impact on thyroid dosing and levothyroxine levels can be further explored. To our knowledge this is the first study to describe the genotype distribution of SNP rs11249460 in the Jordanian population.

Deiodinases on the other hand are extensively studied. Deiodinase 1 (DIO1) was the first to be recognized by biochemical assays of conversion of T4 to T3 and was also the first to be cloned. DIO1 catalyzes T4-to-T3 conversion and supplies a significant fraction of the T3 in plasma of euthyroid humans [22]. In a previous study, the C-allele of SNP rs2235544 in DIO1 was associated with free T3/T4 ratio and an increase in free T3 that reflects increased DIO1 function [11]. Our results confirm this finding in the studied population, the CC allele had the highest mean free T3 levels with decreasing concentrations for the CT then the TT genotypes. Other studies have also confirmed this finding [10, 23]. The association of this SNP with T3 levels was significant after adjusting for multiple testing. In addition this SNP was retained in our regression model as a significant independent predictor of weight-adjusted thyroxine dose, after accounting for other confounders, which essentially can help in personalizing doses for thyroxine replacement in hypothyroid patients by screening for the presence of this SNP in them. Additionally, it was retained in our regression model as a significant independent predictor of T3 levels, but not for T4 nor for TSH. Interestingly, this SNP was also associated with BMI levels. Although this was not significant after adjusting for multiple testing. Studying this SNP can be useful when also studying the added benefit of T3 supplementation in patients with reduced conversion of T4 to T3 whether hypothyroid or in the other diseases where it has been related to such as depression [24]. To our knowledge this is also the first study to describe the genotype distribution and the effects of SNP rs2235544 in the Jordanian population.

Deiodinase 2 (DIO2) catalyzes the conversion of T4 to T3 and rT3 to 3, 3′ T2 [22]. Rs225014 is a SNP in DIO2 that has been previously linked to thyroid hormone metabolism and treatment for hypothyroidism [9, 10, 25]. This SNP was not associated with any of thyroid panel hormones in our study. Although in a study in a Turkish population, it was associated with differences in TSH levels [10], and in another study, it was predictive of levothyroxine dose required to achieve target TSH levels in thyroidectomized patients [13]. The results in our study can potentially be because DIO2 is known to be particularly important in the brain [22] and that levels of the T4 in serum may not reflect actual brain thyroxine levels [25]. The second SNP in DIO2 rs225015 was also not associated with any differences in our study. This SNP was previously correlated to TSH levels in Turkish Population [10]. This is the first study to our knowledge that describes the effects of these two SNPs (Rs225014, rs225015) in the Jordanian population. Previous studies have described other SNPs in DIO2 in the Jordanian population and also found no effect [17].

In addition to deiodination and sulfation, UDP-glucuronyltransferases (UGTs) in the human liver metabolize T4 to thyroxine glucuronide. Previous studies have revealed that T4 glucuronidation is mediated by the UGT1A subfamily enzymes, namely UGT1A1 and UGT1A3 [12, 20, 26–30]. Rs3806596 in UGT1A3 has been studied previously in relation to its effect on thyroid hormones [10, 12]. The TT genotype of this SNP in our study was associated with lower levels of T4, and although it was not significant after adjusting for multiple testing, our result is consistent with the study by Santoro et al. and Arici et al. The C allele was associated with low expression of UGT1A3 which essentially means less T4 is metabolized and serum levels of this hormones are expected to be higher with this allele compared to the T allele [12]. This SNP was retained in our logistic regression model for predicting T4 serum levels after controlling for all other confounders in the same direction. To our knowledge this is also the first study to describe the genotype distribution and the effects of SNP rs3806596 in the Jordanian population.

Rs3064744 in UGT1A1 (previously rs8175347) corresponds to the location of TA repeats in the promoter region which are called *1 (6 repeats), *28 (7 repeats), *36 (5 repeats) and *37 (8 repeats) [31]. The *1 Allele was associated with decreased transcription of UGT1A1 in transfected human hepatoma cell lines as compared to the *28 allele [32]. It also classified into three distinct phenotypes; extensive metabolizer, intermediate metabolizer and poor metabolizer and in all populations studied to date, UGT1A1*1 and UGT1A1*28 are most frequent, while UGT1A1*36 and TA8 UGT1A1*37 repeats are infrequent or absent depending on geographic region of ancestry [33]. In our study we only had the three genotypes: UGT1A1*1, UGT1A1*28 and UGT1A1*36 with only two phenotypes: *1/*28 intermediate metabolizers and *1/*36 extensive metabolizers. In our analysis this SNP was correlated with T4 levels and with BMI. Where the intermediate metabolizers had higher T4 levels and higher BMI compared to extensive metabolizers. This SNP was not retained however in any logistic regression model that controls for confounders. Arici et al. didn’t find an influence of this SNP on thyroid hormone levels while Santoro et al. studied this SNP as part of a haplotype in UGT1A1 [10, 12]. This SNP is in tight linkage disequilibrium (LD) with two other SNPs in UGT1A3 (rs3806596 which we studied here and rs1983023) [12]. And its influence might be nuanced by other SNPs in linkage to it. This SNP frequency has been described in the Jordanian population but not its relation to thyroid hormone level and replacement [34].

Vitamin D has gained attention in recent years, and its deficiency has been correlated to autoimmune thyroid disease. Impaired vitamin D signaling has also been implicated in thyroid tumorigenesis[35]. As a possible influencer on levothyroxine dosage requirements, we studied vitamin D levels, supplementation and two SNPs that were previously correlated to Vitamin D levels. The First SNP we studied was in the RXRA gene. RXRA gene encodes the retinoic X receptor alpha which is also a transcription factor that was found to influence gene expression as a response to thyroid hormone binding to its receptor [36]. Interestingly rs11185644 in this gene was previously found to influence Vitamin D response to supplementation [37]. In our study, we report for the first time that this SNP also influences levothyroxine dosage requirements after controlling for vitamin D serum level and supplementation status and provides a link for the role of Vitamin D in thyroid disease. If confirmed in larger trials, this would influence how hypothyroidism will be treated. To our knowledge this is the first study to report the genotype and effect of SNP rs11185644 in the Jordanian population. The other SNP rs4588 was previously associated with baseline levels of Vitamin D but had no effect on levothyroxine dosage requirement nor thyroid hormone levels in our study. Its genotype frequency and association with the levels of Vitamin D has previously been reported in the Jordanian population [36, 38].

And lastly Vitamin B12 deficiency was associated with autoimmune thyroid disease [39]. The A allele rs602662 in Fucosyl transferase 2 gene (FUT2) was correlated to higher levels of serum Vitamin B12 previously, however this SNP had no correlations in our study with either level of Vitamin B12 nor the thyroid hormones [40]. To our knowledge this is the first study to report the genotype and effect of SNP rs602662 in the Jordanian population.

We understand that our study is not powered to provide recommendations for dose changes in clinical practice, but they highlight potential future ways of calculating and optimizing doses for thyroid patients. The SNPs that we studied could be in linkage to nearby SNPs that may be the real cause of the change. It is also important to acknowledge the limitations of our study. One of these limitations is that we had incomplete information regarding the doses of supplements, especially for Vitamin D. Furthermore, we did not have information on sun exposure which has a role in the synthesis of Vitamin D. Additionally, we did not measure levels of thyrotropin hormone releasing hormone which is also an important component involved in the regulation of thyroid hormones levels. Lastly, our cohort consisted of more females compared to males which may introduce bias. It is worthwhile mentioning here that this study was conducted during the COVID-19 pandemic and associated lockdowns where we had difficulty in recruitment in general.

### Study highlights

#### What is the current knowledge on the topic?

Primary hypothyroidism due to abnormality in the thyroid gland is the most common endocrine disease. Levothyroxine is used as replacement hormone and started at the recommended dose of 1.6 µg/kg. Frequent dosage adjustments are necessary to reach optimum treatment which may influence patients’ quality of life. Many factors determine LT4 dose requirements including genetic polymorphisms. Some of these genetic polymorphisms have been studied in relation to levothyroxine bioavailability but not clearly related to exact dosage needs.

#### What question did this study address?

This study aimed to study the influence of pre-selected and novel candidate polymorphisms on T4 dose requirements.

#### What does this study add to our knowledge?

This study identifies the influence of the SNP rs11185644 in RXRA gene and rs2235544 in DIO1 on dosage requirements and levothyroxine bioavailability in the Jordanian population.

#### How might this change clinical pharmacology or translational science?

If confirmed in a larger group of people, this may change how initial doses of levothyroxine replacement therapy are calculated and will help in translating pharmacogenomics science into practice.

## Data Availability

Datasets analyzed during the current study are available in the figshare repository by clicking the following link 10.6084/m9.figshare.22059068. Data from this study have also been submitted to ClinVar database https://www.ncbi.nlm.nih.gov/clinvar/ and are searchable with the following accession numbers: SCV004020289 (rs11249460), SCV004020290 (rs2235544), SCV004020291 (rs225014), SCV004020292 (rs225015), SCV004020293 (rs3806596), SCV004020294 (rs3064744), SCV004020295 (rs11185644), SCV004020296 (rs4588) and SCV004020297 (rs602662).
